# B-1a B Cells Regulate T Cell Differentiation Associated with Pregnancy Disturbances

**DOI:** 10.3389/fimmu.2014.00006

**Published:** 2014-01-21

**Authors:** Damián Oscar Muzzio, Rocío Soldati, Luise Rolle, Marek Zygmunt, Ana Claudia Zenclussen, Federico Jensen

**Affiliations:** ^1^Department of Experimental Obstetrics and Gynecology, Medical Faculty, Otto-von-Guericke University, Magdeburg, Germany; ^2^Research Laboratory, Department of Obstetrics and Gynecology, University of Greifswald, Greifswald, Germany

**Keywords:** B-1a B cells, pregnancy, Th17, Th1, tolerance

## Abstract

During pregnancy, the maternal immune system faces a double dilemma: tolerate the growing semi-allogeneic fetus and at the same time protect the mother and the progeny against pathogens. This requires a fine and extremely regulated equilibrium between immune activation and tolerance. As professional antigen presenting cells, B cells and in particular B-1a B cells, can activate or tolerize T cells and thus participate in the generation or regulation of the immune response. B-1a B cells were involved in the humoral immune response leading to pre-eclampsia, one of the main medical complications during pregnancy. Here we demonstrated that B-1a B cells are additionally involved in cellular immune mechanisms associated with pregnancy complications. Using a mouse model of pregnancy disturbances, we showed that B-1a B cells from animals suffering pregnancy disturbances but not from those developing normal pregnancies induce the differentiation of naïve T cells into Th17 and Th1 cells. This differential role of B-1a B cells during pregnancy seems to be associated with the co-stimulatory molecule CD86 as normal pregnant mice showed lower percentages of CD86 expressing B-1a B cells as compared to pregnant mice developing pregnancy disturbances or to non-pregnant animals. Our data bring to light a new and not explored role of B-1a B cells in the context of pregnancy.

## Introduction

Pregnancy in eutherian mammals represents a unique and fascinating immunological process in which paternal, semi-allogeneic, antigens carried by the fetus must to be “tolerated” by the maternal immune system (1). This requires a fine and highly regulated balance between immune activation, to ensure proper protection of the mother against pathogens, and immune tolerance that allows the survival of the semi-allogeneic fetuses within the maternal uterus. Minimal disturbances of this fine equilibrium may have deleterious effect not only in the progeny but also in the mother (1).

Paternal antigens, present in the semen, are being recognized by the maternal immune system even before the process of fecundation has taken place (2, 3). As pregnancy is established, embryo starts growing and the transfer of fetal cells, as well as cell debris into the maternal circulation increases (4). As for other antigens, paternal antigens can be up-taken by maternal professional antigen presenting cells (APCs), processed and presented to naïve T cell encouraging their differentiation into different T cell lineages, namely: Th1, Th2, Th17, or regulatory T cells depending on different signals (2, 5–7).

Pregnancy wellbeing was classically associated with a shift toward a Th2 profile while the prevalence of Th1 as well as Th17 cells is associated with different pregnancy-associated pathologies, e.g., miscarriages (8, 9), intrauterine growth restriction (10), pre-eclampsia (11, 12), and pre-term birth (13, 14).

Among professional APCs, peritoneal cavity (PerC) B-1a B cells have been shown to be potent inducers of Th1 (15) as well as Th17 T cell differentiation (16). However, under especial conditions, e.g., CD86 blockage, B-1a B cells fail to induce Th1/17 T cell polarization. Instead, they strongly boost the differentiation of naïve T cells into newly expressing FOXP3 inducible regulatory T cells (iTregs) (15).

We have previously demonstrated that B-1a B cells are involved in the humoral immune response associated with the physiopathology of pre-eclampsia, one of the leading causes of pregnancy complications (17). In this study we aimed to further investigate the capacity of B-1a B cells to regulate the differentiation of the pro-inflammatory Th17 and Th1 T cells subsets in a murine model of pregnancy disturbances.

## Materials and Methods

### Animals and animal model of pregnancy disturbances

Eight weeks old CBA/J (H2^k^) and C57BL/6 (H2^b^) females as well as BALB/c (H2^d^) and DBA/2J (H2^d^) males were purchased from Charles River (Germany/France). Mice were kept in our animal facility under optimal conditions in a 12-h light cycle. Chow and water were applied *ad libitum*. Animal experiments were carried out according to institutional guidelines after Ministerial approval [Reviewing board institution: Landesverwaltungsamt Sachsen-Anhalt (ID: FJ2-101 to Federico Jensen) and Landensamt für landwirtschaft Lebensmittelsicherheit und Fischrei Mecklenburg-Vorpommer (7221.3-1-068/13 to Federico Jensen)]. The experiments were conducted in conformity with the European Communities Council Directive.

A well-established model of pregnancy disturbances was used (18). Briefly, when CBA/J females are mated to DBA/2J males, a median of 20–30% of the embryos resorption (abortions) is observed (19). Besides, pregnant females from this combination are also characterized by angiogenic deregulation, abnormal placental development, and fetal growth restriction, all features of pre-eclampsia (20). BALB/c mated CBA/J females represent the normal pregnancy combination with a median of 0% of abortions and none of the symptoms described above for the DBA/2J mated CBA/J females.

Virgin females CBA/J mice were mated with BALB/c, or DBA/2J males. Females were inspected daily for vaginal plugs, and presence of a vaginal plug was designated as day 0 of pregnancy. Pregnant females were sacrificed at day 14 of pregnancy.

### Antibodies and reagents

The following anti-mouse fluorescently labeled antibodies were used: CD19 (clone 1D3), CD11b (clone M1/70), CD5 (clone 53-7.3), CD80 (clone 16-1OA1), MHCII (clone 2G9), CD86 (clone GL1) (BD Biosciences, Germany), and CD23 (clone B3B4) (eBiosciences, Germany). Mitomycin-*c* was obtained from Sigma-Aldrich, Germany. CD19 MicroBeads isolation kit, CD5 Microbeads isolation kit, and regulatory T cells isolation kit were obtained from Miltenyi Biotec, Germany. Anti-mouse IL4 and anti-mouse IFNγ were from BD, Biosciences, Germany. TGFβ was purchased from R&D System, Germany. IL23 and IL6 were obtained from eBiosciences, Germany. Cytometric bead array (CBA) Mouse Th1/Th2/Th17 Cytokine Kit was obtained from BD, Biosciences, Germany.

### Cell isolation and culture

CD19^+^CD5^+^ B-1a B cells were magnetically isolated from PerC washouts of BALB/c or DBA/2J mated CBA/J pregnant females on day 14 of pregnancy. As control B-1a B cells were isolated from non-pregnant CBA/J females. Pure isolated B-1a B cells were treated with mitomycin-*c* and used as APCs. CD4^+^CD25^−^ naïve T cells were isolated from lymph nodes of non-pregnant C57BL/6 females. Isolated naïve T cells (2 × 10^5^) were cultured with mitomycin-*c* inactivated B-1a B cells (1 × 10^5^) (2:1) in 96-well round-bottom plates with 200 μl of RPMI medium supplemented with SFB (10%) and antibiotics for 5 days with or without the addition of a Th17 differentiation cytokines cocktail (10) composed of anti IFNγ (10 μg/ml), anti IL4 (10 μg/ml), TGFβ (3 ng/ml), IL6 (50 ng/ml), and IL23 (20 ng/ml). Supernatants were collected and frozen at −80°C.

### Cell staining and flow cytometry

Peritoneal cavity cells were stained with specific antibodies or matched isotype controls for 30 min at 4°C. After washing, cells were analyzed with a FACSCalibur flow cytometer. Data were analyzed with FlowJo software (Tree Star Inc.). For analyzing the expression levels of MCHII, CD80, CD86, PD-L1, PD-L2, and FASL on CD19^+^CD23^−^CD5^+^ B-1a B cells mean fluorescence index (MFI) was applied using FlowJo software (Tree Star Inc.).

### Cytokine detection in supernatants

Levels of IL17, TNFα, IFNγ, IL2, and IL6 cytokines were measured in supernatants by CBA Mouse Th1/Th2/Th17 Cytokine Kit and Th1/Th2 Inflammation Kit from BD Biosciences, following supplier recommendation. MCP1 was measured by using an ELISA kit from R&D System.

### Statics

The statistical significance of comparisons of median values was assessed by the non-parametric Kruskal–Wallis test with GraphPad software.

## Results

### B-1a B cells from pregnant animals suffering pregnancy disturbances induce Th17 T cell differentiation while B-1a B cells from normal pregnant mice strongly inhibited it

**Figure 1:**
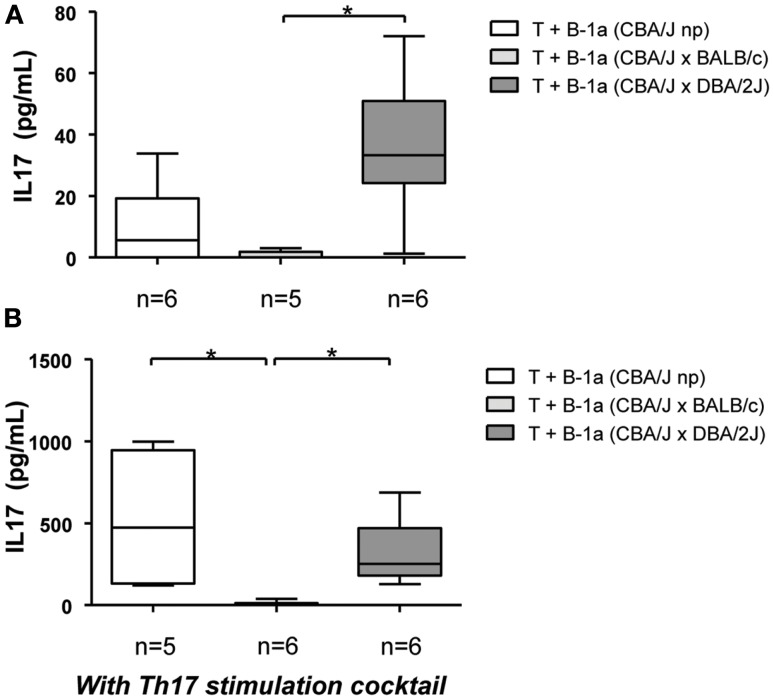
**B-1a B cells from normal pregnant animals but not from pregnant animals suffering pregnancy disturbances inhibit Th17 T cell differentiation**. CD4^+^CD25^−^ naïve T cells (2 × 10^5^) were cultured with mitomycin-*c* treated CD19^+^CD5^+^ B-1a B cells (1 × 10^5^) (1:2) from non-pregnant, BALB/c mated (normal pregnant) or DBA/2J mated (developing pregnancy disturbances) CBA/J females without **(A)** or with **(B)** the addition of a Th17 cytokine differentiation cocktail. Levels of IL17 were assayed in supernatants. Data are expressed as Box and Whisker plots showing median. *P < 0.05 as analyzed by the non-parametric Kruskal–Wallis test.

Increasing evidence indicates that pregnancy disturbances, e.g., unexplained recurrent miscarriages (8) and pre-eclampsia (11) are associated with a prevalence of Th17 cells. B-1a B cells are potent inducers of Th17 cells differentiation (15, 16, 21, 22). Taking these into account we aimed to explore here the differential capacity of B-1a B cells from pregnant mice developing normal pregnancies or mice suffering from pregnancy disturbances to induce Th17 cell differentiation *in vitro*. Mitomycin-*c* treated B-1a B cells were co-cultured with allogeneic CD4^+^CD25^−^ naïve T cells and the production of IL17 was assayed in supernatants. In agreement with previous studies (16), B-1a B cells isolated from non-pregnant virgin control mice induced a slight production of IL17 by CD4^+^CD25^−^ naïve T cells (Figure 1A). This modest production of IL17 was lowered when T cells were cultured with B-1a B cells isolated from normal pregnant mice, although differences did not reach statistical significance (Figure 1A). Interestingly, when B-1a B cells isolated from animals suffering pregnancy disturbances were used to stimulate T cells, a significantly higher production of IL17 was observed as compared to T cells cultured with B-1a B cells from normal pregnant mice (Figure 1A). No differences were observed on IL17 production by T cells cultured with B-1a B cells from non-pregnant mice compared to T cells cultured with B-1a B cells from animal suffering from pregnancy disturbances (Figure 1A).

Differentiation of Th17 cells can be induced *in vitro* by exposing CD4^+^ T cells to optimal conditions of TGFβ, IL6, IL23, and blocking IFNγ and IL4 (16). We next wondered whether B-1a B cells from animals developing normal pregnancies not only fail to induce IL17 production by T cells as compared to B-1a B cells from animals suffering pregnancy disturbances but also can inhibit Th17 differentiation even under optimal conditions for Th17 development. We cultured CD4^+^CD25^−^ naïve T cells with B-1a B cells isolated from pregnant mice developing normal pregnancies with the addition of Th17 optimal condition cytokines. In addition, T cells were cultured with B-1a B cells from pregnant mice suffering pregnancy complications or from non-pregnant mice plus cytokines. As expected, when CD4^+^CD25^−^ naïve T cells were co-cultured with B-1a B cells isolated either from non-pregnant mice or pregnant mice developing pregnancy disturbances and cytokines were added, a potent induction of IL17 production by naïve T cells was observed (Figure 1B). Notably, the production of IL17 by T cells in these two groups was ≈10-fold higher as compared to the same groups without the addition of the Th17 stimulation cocktail (Figure 1A). This clearly confirms the robustness of the Th17 induction system. Remarkably, B-1a B cells isolated from pregnant mice developing normal pregnancies strongly inhibited the production of IL17 by CD4^+^CD25^−^ naïve T cells even under the addition of optimal cytokines conditions for Th17 cell development (Figure 1B). These results clearly demonstrate a differential capacity of B-1a B cells in the context of pregnancy: while B-1a B cells from animals developing pregnancy disturbances induce Th17 cells polarization, B-1a B cells from animals undergoing normal pregnancies manifested a strong inhibitory capacity.

### B-1a B cells from pregnant animals suffering pregnancy disturbances but not from those developing normal pregnancies induced the production of Th1 cytokines by T cells

**Figure 2:**
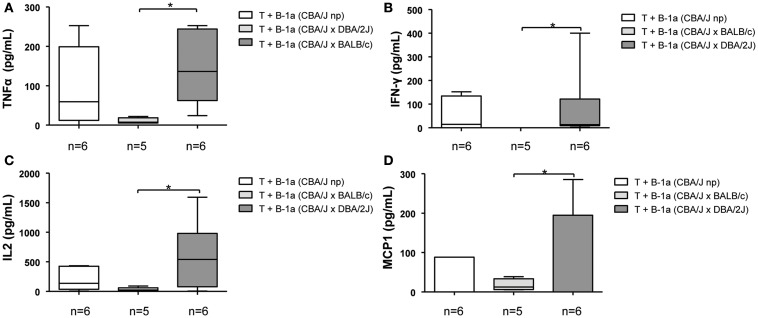
**B-1a B cells from pregnant animals developing pregnancy complications but not from those having normal pregnancies induce Th1 T cell polarization**. CD4^+^CD25^−^ naïve T cells (2 × 10^5^) were cultured with CD19^+^CD5^+^ B-1a B cells (1 × 10^5^) (2:1) from non-pregnant, BALB/c (normal pregnant) or DBA/2J (developing pregnancy disturbances) pregnant CBA/J females. Levels of TNFα **(A)**, IFNγ **(B)**, IL2 **(C)**, and MCP1 **(D)** Th1 cytokines were analyzed in supernatants. Data are expressed as Box and Whisker plots showing median. *P < 0.05 as analyzed by the non-parametric Kruskal–Wallis test.

In addition to their capacity to induce Th17 differentiation, B-1a B cells were also shown to participate in the induction of Th1 polarization (15). We next wondered whether in the context of pregnancy, B-1a B cells might also have a differential role in inducing/inhibiting Th1 polarization. We observed that mitomycin*-c* treated B-1a B cells isolated from normal pregnant mice induced a lower but not significant production of Th1 pro-inflammatory cytokines, TNFα, IFNγ, IL2, and MCP1 by CD4^+^CD25^−^ naïve T cells as compared to B-1a B cells from non-pregnant control animals (Figures 2A–D). However, when T cells were cultured with B-1a B cells isolated from pregnant mice developing pregnancy disturbances, significantly higher levels of Th1 pro-inflammatory cytokines were observed as compared to T cells cultured with B-1a B cells isolated from normal pregnant mice (Figures 2A–D). No differences were observed on Th1 cytokines production by T cells cultured with B-1a B cells from non-pregnant mice compared to T cells cultured with B-1a B cells from animal suffering from pregnancy disturbances (Figures 2A–D). These results demonstrate that B-1a B cells from pregnant mice developing normal pregnancies induced a significantly lower production of Th1 cytokines by T cells as compared to B-1a B cells from animals suffering pregnancy disturbances.

### Levels of B-1a B cells in PerC do not change during pregnancy

**Figure 3:**
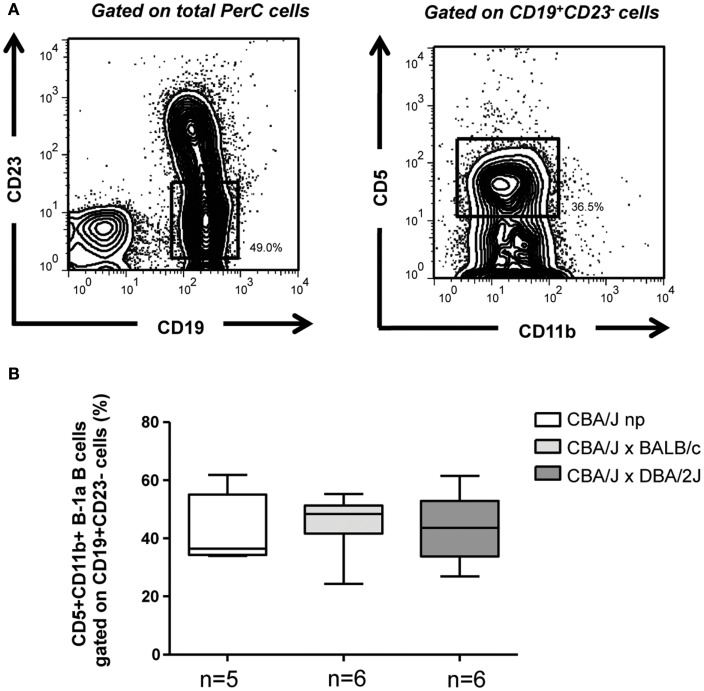
**Percentages of B-1a B cells do not change during pregnancy**. **(A)** Representative counter plots displaying the gating strategy used to analyze the percentages of CD19^+^CD23^−^CD11b^+^CD5^+^ B-1a B cells in the PerC of non-pregnant and pregnant mice. **(B)** Graph displays percentages of CD19^+^CD23^−^CD11b^+^CD5^+^ B-1a B cells in the PerC of non-pregnant as well as BALB/c (normal pregnant) and DBA/2J (pregnancy disturbances) pregnant CBA/J females. Data are expressed as Box and Whisker plots showing median. Differences were analyzed by the non-parametric Kruskal–Wallis test.

**Figure A1:**
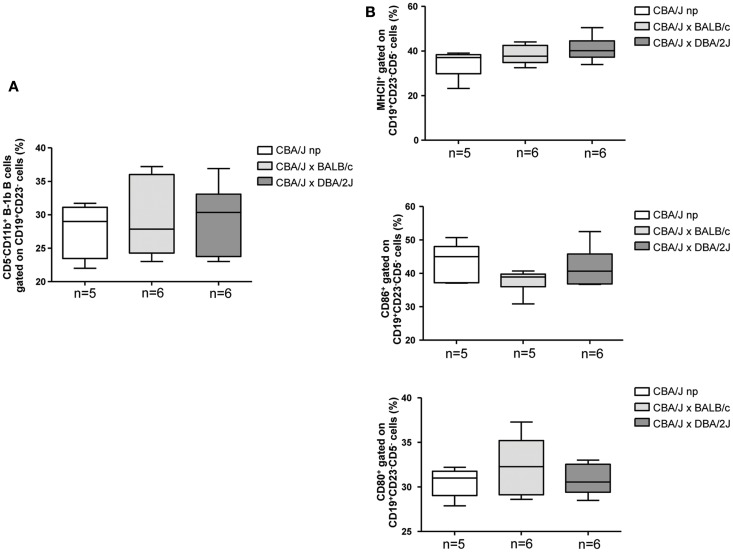
**Percentages of B-1b cells as well as percentages of MHCII, CD86, and CD80 expressing B-1b cells are similar in normal pregnant mice and in pregnant mice suffering pregnancy disturbances**. **(A)** Graph showing no differences in PerC CD19^+^CD23^−^CD11b^+^CD5^−^B-1b cells percentages between non-pregnant and normal pregnant mice or pregnant mice suffering pregnancy disturbances. **(B)** Percentages of MHCII, CD80, and CD86 expressing PerC CD19^+^CD23^−^CD5^−^B-1b cells are similar among the groups.

We next concentrated in the mechanisms behind the differential capacity of B-1a B cells from pregnant animals having normal pregnancies and those developing pregnancies disturbances in the regulation of Th1/17 T cell differentiation. We began analyzing the percentages of CD19^+^CD23^−^CD11b^+^CD5^+^ B-1a B cells (23) in the PerC of pregnant and non-pregnant mice. As displayed in Figure 3 no differences were observed concerning the percentages of CD19^+^CD23^−^CD11b^+^CD5^+^ B-1a B cells in the PerC of pregnant animals having normal pregnancies compared to those suffering pregnancy disturbances and to non-pregnant controls. We have additionally analyzed the percentages of CD19^+^CD23^−^CD11b^+^CD5^−^B-1b cells as well as CD19^+^C23^+^ B2 B cells. Neither the percentages of B-1b cells (Figure A1A in Appendix) nor percentages of B2 B cells (data not showed) in PerC of non-pregnant as well as normal pregnant mice or animals suffering pregnancy disturbances were modified. These results suggest that the differential capacity of B-1a B cells from normal pregnant mice in inhibiting Th17/1 T cell differentiation compared to B-1a B cells from animal suffering pregnancy disturbances, is not related to changes in the levels of B-1a B cells.

### Pregnant animals developing normal pregnancies and animals suffering pregnancy disturbances have similar levels of MHCII expressing B-1a B cells

**Figure 4:**
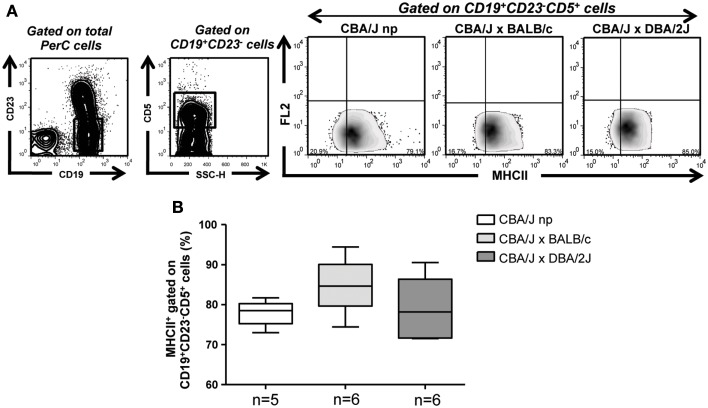
**B-1a B cells from pregnant animals developing normal pregnancies and animals suffering pregnancy disturbances display similar levels of MHCII expressing B-1a B cells**. **(A)** Representative contour and density plots displaying the gating strategy used to analyze the percentages of MHCII expressing CD19^+^CD23^−^CD5^+^ B-1a B cells in the PerC of non-pregnant and pregnant mice. **(B)** Graph depicts percentages of MHCII expressing CD19^+^CD23^−^CD5^+^ PerC B-1a B cells from non-pregnant as well as BALB/c (normal pregnant) and DBA/2J (pregnancy disturbances) pregnant CBA/J females. Data are expressed as Box and Whisker plots showing median. Differences were analyzed by the non-parametric Kruskal–Wallis test.

B cells present antigens to T cells in the context of MHCII molecule (24, 25). Having observed that percentages of B-1a B cells do not differ between animals having normal pregnancies compared to those developing pregnancy disturbances or to non-pregnant mice, we next analyzed the expression levels of MHCII molecule on CD19^+^CD23^−^CD5^+^ B-1a B cells as well as the percentages of MHCII expressing CD19^+^CD23^−^CD5^+^ B-1a B cells in non-pregnant as well as pregnant mice having normal pregnancies or developing pregnancies disturbances. Neither the expression levels of MHCII in CD19^+^CD23^−^CD5^+^ B-1a B (MFI: 2.7, 2.8, and 2.4, respectively) nor the percentages of MHCII expressing CD19^+^CD23^−^CD5^+^ B-1a B cells in PerC were modified among the groups (Figure 4). Similar results were observed when analyzing MHCII expression on CD19^+^CD23^−^CD5^−^B-1b cells (Figure A1B in Appendix)

### Percentages of CD86 expressing B-1a B cells are diminished during normal pregnancy

**Figure 5:**
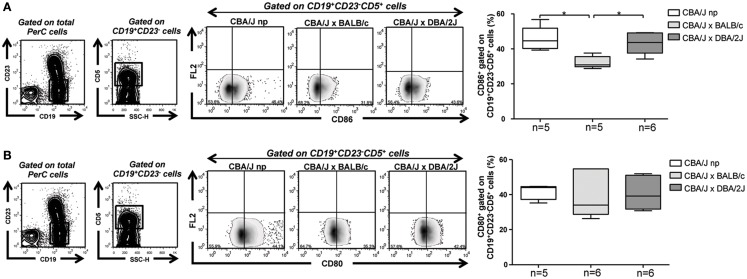
**CD86 expressing B-1a B cells are diminished in pregnant mice developing normal pregnancies but not in those suffering pregnancy disturbances**. **(A)** Representative contour and density plots depicting gating strategy used to quantify percentages of CD86 expressing CD19^+^CD23^−^CD5^+^ PerC B-1a B cells of non-pregnant as well as BALB/c (normal pregnant) and DBA/2J (pregnancy disturbances) pregnant CBA/J females. Graph shows percentages of CD86 expressing CD19^+^CD23^−^CD5^+^ PerC B-1a B cells. **(B)** Representative contour and density plots depicting gating strategy used to quantify the percentages of CD80 expressing CD19^+^CD23^−^CD5^+^ PerC B-1a B cells of non-pregnant as well as BALB/c (normal pregnant) and DBA/2J (pregnancy disturbances) pregnant CBA/J females. Graph shows percentages of CD80 expressing CD19^+^CD23^−^CD5^+^ PerC B-1a B cells from non-pregnant as well as BALB/c or DBA/2J mated CBA/J females. Data are expressed as Box and Whisker plots showing median. *P < 0.05 as analyzed by the non-parametric Kruskal–Wallis test.

Activation and differentiation of naïve T cells upon antigen presentation require a proper interaction of CD28 expressed on T cells with co-stimulatory molecules (CD86 and CD80) expressed on APC (26–28). We observed similar levels of CD80 and CD86 expression on PerC CD19^+^CD23^−^CD5^+^ B-1a B cells from non-pregnant mice compared to pregnant mice developing normal, pregnancies or suffering pregnancy disturbances (CD80 MFI: 2.2, 2.2, and 2.5; CD86 MFI: 2.3, 2.1, and 2.4, respectively). However, percentages of CD86 expressing CD19^+^CD23^−^CD5^+^ B-1a B cells were significantly lower in PerC of pregnant mice developing normal pregnancies as compared to those suffering pregnancy disturbances or to non-pregnant control mice (Figure 5A). No differences were observed concerning percentages of CD80 expressing CD19^+^CD23^−^CD5^+^ B-1a B cells among the groups (Figure 5B). We have also analyzed the percentages of CD86 and CD80 expressing CD19^+^CD23^−^CD5^−^B-1b cells in PerC of non-pregnant as well as normal pregnant or pregnant mice suffering pregnancy disturbances and did not find any difference among the groups (Figure A1B in Appendix).

**Figure A2:**
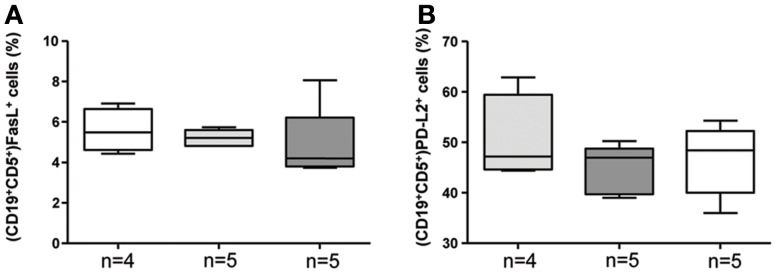
**Levels of FASL and PD-L2 expressing B-1a B cells are not affected in normal as well as disturbed pregnancies**. Levels of FASL **(A)** and PD-L2 **(B)** expressing CD19^+^CD23^−^CD5^+^ PerC B-1a B cells are similar between non-pregnant and pregnant mice.

We have additionally studied the expression levels of cellular markers (PD-L1, PD-L2, and FASL) on CD19^+^CD23^−^CD5^+^ B-1a B cells, all well known to take part in the interaction between B-1a and T cells. As for other cellular markers analyzed in this study we did not find any difference in the expression levels of PD-L1 (MFI: 318.5; 327 and 310, respectively), PD-L2 (MFI: 145, 138, and 141, respectively), and FASL (MFI: 36.70, 30.80, and 30.40, respectively) as well as in the percentages of PD-L1 (100% of B-1a B cells of all groups expressed PD-L1, data not showed), PD-L2 and FASL expressing CD19^+^CD23^−^CD5^+^ B-1a B cells between the groups (Figure A2 in Appendix). These results suggest that the lower percentages of CD86 expressing B-1a B cells observed in normal pregnant animals may be one of the mechanisms as how these cells fails to induce Th1 and strongly inhibit Th17 T cell differentiation during normal pregnancy.

## Discussion

The ability of B-1 B cells to induce the polarization of naïve T cells into pro-inflammatory Th17 and Th1 T cell subsets has been extensively demonstrated (15, 16, 21, 22). In this study we have confirmed and extended these results by showing that in the context of pregnancy B-1a B cells display a differential capacity in term of regulating pro-inflammatory T cell differentiation. B-1a B cells from pregnant mice developing normal pregnancies not only failed to induce Th17 differentiation but also showed to have a strong inhibitory capacity even in a favorable environment for the differentiation of Th17 cells. Notably, this inhibitory effect was not achieved when B-1a B cells were isolated from pregnant animals suffering pregnancy disturbances, clearly demonstrating a differential capacity of B-1a B cells from normal pregnant animals in controlling Th17 T cell differentiation.

Similarly, B-1a B cells from pregnant animals suffering pregnancy disturbances but not from animals developing normal pregnancies induced the production of pro-inflammatory Th1 cytokines by naïve T cells. These results suggest that during normal pregnancy B-1a B cells are turned into an transient state of tolerance that unable them to induce pro-inflammatory T cell activation and differentiation that can be deleterious for pregnancy to be maintained (29).

Activation of naïve T cells and posterior maturation into different T cell lineages is a multistep phenomenon requiring both, antigen-specific triggering of the T cell receptor complex and additional signaling via co-stimulatory molecules (26, 27). B cell antigen presentation to T cells occurs in the context of MHCII molecule and the interaction of CD28 receptor expressed on T cells with CD86 and CD80 ligands expressed on the B cells (24, 28). Blockage of CD28 and their ligands, CD86 (30) and CD80, has been shown to induce antigen-specific peripheral tolerance (15). In this regards, we showed a significant reduction in the percentages of CD86 expressing B-1a B cells in PerC of animals developing normal pregnancies as compared to non-pregnant mice and to pregnant mice suffering pregnancy disturbances. To note is the fact that despite the observed differences in the percentages of CD86 expressing B-1a B cells, the expression levels of CD86 in B-1a B cells, analyzed by MFI, was similar between the groups. Based on our results it is tempting to speculate that the reduction in the percentages of CD86 expressing B-1a B cells rather than down-regulation of CD86 itself on B-1a B cells, represents an adaptive mechanisms aimed to avoid undesired activation of pro-inflammatory T cells that can have a deleterious effect on pregnancy outcome. Interestingly, using the same animal model of pregnancy disturbance we used in this study, Jin and co-authors have showed that blocking CD86 with specific antibody, considerably improved pregnancy outcome in pregnant mice otherwise undergoing pregnancy disturbances (30, 31). In addition, Wang and Rothstein using either a CD86 neutralizing antibody or B-1a B cells isolated from CD86 KO animals have recently showed that the lack of CD86 expression on B-1a B cells is linked to their incapacity to induce Th17 differentiation (16). In keeping with our results, Zhong and co-authors have nicely demonstrated that the ability of B-1a B cells to stimulate alloreactive T cells can be inhibited by blocking CD86 expression on B-1a B cells, rather under this condition, B-1a B cells favor the development of inducible FOXP3 newly expressing regulatory T cells (iTregs) (15).

In conclusion, we have demonstrated in this study that B-1a B cells manifest a differential capability in terms of regulating pro-inflammatory T cell activation and differentiation during pregnancy. Besides, we provided evidences that this differential capacity may be associated with the levels of CD86 expressing B-1a B cells.

Overall, we provided evidences highlighting the central role of B-1a B cells in controlling pro-inflammatory T cell activation and polarization in the context of pregnancy. This opens a new avenue for exploring strategies to control undesired immune activation that may compromise pregnancy outcome.

## Author Contributions

Damián Oscar Muzzio performed experiments and analyzed data. Rocío Soldati and Luise Rolle performed experiments. Ana Claudia Zenclussen and Marek Zygmunt contributed with reagents and with the design of the experiments. Federico Jensen designed experiments, analyzed data, contributed with reagents, wrote the paper, and supervised the work.

## Conflict of Interest Statement

The authors declare that the research was conducted in the absence of any commercial or financial relationships that could be construed as a potential conflict of interest.
